# It’s Time to Move Beyond Traditional Health Care Worker Training Approaches

**DOI:** 10.9745/GHSP-D-21-00553

**Published:** 2021-09-30

**Authors:** Julia Bluestone, Jim Ricca, Denise Traicoff, Dieula Delissaint Tchoualeu

**Affiliations:** a Jhpiego, Baltimore, MD, USA.; b Global Immunization Division, Center for Global Health, U.S. Centers for Disease Control and Prevention, Atlanta, GA, USA (Retired).; c Global Immunization Division, Center for Global Health, U.S. Centers for Disease Control and Prevention, Atlanta, GA, USA.

## Abstract

Isn't it time that the global community move beyond traditional training and supportive supervision models to improve health care worker capacity?

See related article by Traicoff et al. and Tchoualeu et al.

Increasingly, donors and ministry of health officials are recognizing that historical approaches to training and supervision have not resulted in desired changes in provider performance, quality of care, and improved health outcomes. The traditional, classroom-based trainer of trainer (TOT) and cascade approach evolved in an era when access to knowledge was limited to a small set of individuals, or master trainers, and the process of bringing individuals into an off-site, hotel-based location catered to the logistical convenience of international or regional trainers. Evidence has accumulated that such approaches yield disappointing results. A 2016 review of national surveys in sub-Saharan Africa found that these traditional interventions were associated with only modest improvements—^1^

*equivalent to 2 additional provider actions out of the 18–40 actions expected per visit.*

At the same time, evidence has shown that there are approaches that deliver training more effectively and efficiently than the classic group-based training, which removes health care workers from the workplace, and that instead develop their skills in the workplace itself.^2^ Consequently, emphasis has increased on workplace-based training combined with mentorship and follow-up. Such approaches have been facilitated through expanded access to digital technology and real-time data to support just-in-time mobile learning.

This issue of *Global Health: Science and Practice* has 2 articles from Traicoff et al.^3^ and Tchoualeu et al.^4^ on an intervention for improving knowledge and practices of immunization programming in 3 regions of Ghana. The authors of these articles would like to reflect on increasing the effectiveness and rigorous evaluation of cascade training models. These articles describe the methods and results of a multipronged effort that relied on cascade training. Traicoff et al.^3^ point out the inherent risks of TOT, including but not limited to a lack of resources or planning for the cascade. They describe their efforts to mitigate the risks via design, delivery, and post-training support. One divergence of the approach from the traditional cascade model was that the trainers were trained on the training role itself and on adult learning methods. All too often, in traditional cascade training models, trainers are assumed to have the requisite abilities and attitudes needed to carry out training and are simply trained on the learning content. Also, trainers in the program were given clear expectations from management, action planning, mentoring, and several job aids to assist them as they cascaded the training. Still, a majority of their interventions were in classroom rather than workplace settings (“65 workshops, 43 field visits, and 4 review meetings, reaching 1,378 HCWs within 7 months”^3^).

The results from these mid-scale interventions seemed to have been incrementally better than those that could be expected from the traditional approach. Tchoualeu et al. wrote^4^:

*Modest but not statistically significant improvements were found in knowledge on [Expanded Programme on Immunization] policy, immunization data management, and communication skills with caregivers. Health care workers reported that they had improved several attitudes and practices after the [Second Year of Life] training. The most improved practice reported by [health care workers] and observed in all 3 regions was the creation of a defaulter list.*

While these interventions were not shown to be statistically significant, qualitative data were informative to determine the impact of the trainings on changes in provider behaviors.

**Still, we should ask ourselves “isn’t it time that we, as a global community, move even more boldly beyond traditional training and supportive supervision models?”** If so, what would that look like? A 2013 literature review on effective in-service training techniques, setting, frequency, and media found that interactive, case-based learning, hands-on practice or simulation, delivered in the workplace can improve learning outcomes; and that computer or mobile-delivered instruction, if appropriately designed for user engagement, can be equally as effective as live instruction.^5^ In 2009, Rowe et al. published a landmark literature review on interventions to improve health care provider performance in low- and middle-income countries. This review concluded that “training alone results in low effect size.”^6^ These findings support the extensive research that has been done in the field of human performance technology, which confirms the relatively low impact of training compared to the environmental factors affecting a worker.^7^ Rowe recently published an update, using an expansion of the database that he and his collaborators had established that now includes data from 199 studies from 51 countries. This update concluded that educational outreach visits to facilities^*^ (i.e., mentoring visits) were somewhat more effective than in-service training and that in-service training effectiveness was greater if it included clinical practice, occurred within the workplace, and was combined with supervision.^8^ Not only can experiential, on-site approaches be more effective in improving learner and clinical outcomes, they also reduce health worker absenteeism and disruption of services.

Significant challenges remain in terms of operationalizing these newer approaches to improving health provider capacity, especially at scale and under routine conditions. During this transition period, the practical tools and evaluation described in these articles can be useful to training programs. To move forward, sustainable mechanisms need to be built into systems to give providers protected time to learn new or additional skills. Managers can use Gottfredson and Mosher's risk analysis model to identify those skills that carry the greatest risk of harm if done improperly and prioritize them for supervision and workplace support.^2^ There are also questions about how best to ground these efforts to support and improve health care provider performance in more comprehensive quality improvement initiatives. There are ample opportunities to leverage technology to support effective learning and more cost-effective and targeted supervision. The coronavirus disease (COVID-19) pandemic has increased access and acceptability of distance-based delivery methods and has provided opportunities for innovative training, peer learning, and supportive supervision. Whether providing the means for just-in-time learning moments, virtual mentorship visits, facilitation of targeted in-person supervision based on performance, or real-time monitoring of data on key indicators via dashboards, the opportunities to thoughtfully integrate appropriate and sustainable digital solutions have never been greater. Finally, there is a need to commit to evaluating the learning experience and tracking outcomes, including emphasizing qualitative data analysis and measuring informal learning.^9^ A fresh approach to training design, workplace support, and independent evaluation can support accountability and help public health programs develop a competent, confident, and continually improving workforce.

## Footnotes

* Rowe et al. define education outreach visits to facilities as an “on-the-job training strategy with face-to-face visits to individual health care providers at their workplace by persons who health care providers regard as an expert or opinion leader to promote best practices. Also known as academic detailing.”

## Notes

**Peer Reviewed**
